# Area-Level Rurality and Documented Musculoskeletal Symptom Presentation at Rheumatoid Arthritis Diagnosis Among Older Adults in Rural Japan: A Cross-Sectional Study

**DOI:** 10.3390/healthcare14203453

**Published:** 2026-10-15

**Authors:** Ryuichi Ohta, Kunihiro Ichinose, Chiaki Sano

**Affiliations:** 1 Department of Community Care, Unnan City Hospital, Unnan 699-1221, Japan; 2 Department of Rheumatology, Faculty of Medicine, Shimane University, Izumo 693-8501, Japan; kichinose@med.shimane-u.ac.jp; 3 Community Medicine Management, Faculty of Medicine, Shimane University, Izumo 693-8501, Japan; sanochi@med.shimane-u.ac.jp

**Keywords:** rheumatoid arthritis, rural health, older adults, healthcare access, diagnostic pathway, Rurality Index for Japan

## Abstract

**Background:** Recognizing rheumatoid arthritis (RA) in older adults is challenging because clinical presentations are heterogeneous and musculoskeletal symptoms may be attributed to aging or other conditions. Rural context may influence symptom recognition, healthcare-seeking, and diagnostic pathways. We examined whether area-level rurality was associated with musculoskeletal symptoms documented at RA diagnosis among older adults in rural Japan. **Methods:** We conducted a cross-sectional analysis of 221 patients aged ≥65 years diagnosed with RA at a rural community hospital in Japan between 2016 and 2021. Area-level rurality was measured using the Rurality Index for Japan (RIJ). The primary outcome was documentation of hand, wrist, foot, or ankle symptoms at diagnosis. Logistic regression was used to examine the association between RIJ and the primary outcome. The primary adjusted model included age, sex, and Charlson Comorbidity Index. **Results:** Among 221 patients, 106 (48.0%) had documented hand, wrist, foot, or ankle symptoms. RIJ scores were higher among patients with these documented symptoms than among those without them (70.0 vs. 64.8; *p* = 0.004), and the interval from symptom onset to the study-hospital consultation was longer (48.5 vs. 28.0 days; *p* < 0.001). Each 10-point increase in RIJ was associated with higher odds of documented hand, wrist, foot, or ankle symptoms in the primary adjusted model (OR 1.30, 95% CI 1.07–1.57; *p* = 0.008). Exploratory sequential models incorporating consultation timing, CRP, and anti-CCP antibody levels yielded similar estimates. Sensitivity analysis accounting for clustering by residential postal-code area did not materially alter the association. **Conclusions:** Greater area-level rurality was modestly associated with the joint symptom pattern documented at RA diagnosis among older adults in this rural healthcare system. The findings should be interpreted as reflecting differences in documented clinical presentation within a diagnostic pathway rather than as evidence of a distinct rural RA phenotype. In rural settings, clinicians should systematically assess musculoskeletal symptoms in older adults with suspected inflammatory arthritis while remaining attentive to atypical presentations. Prospective multicenter studies using standardized joint assessments and direct individual-level measures of healthcare accessibility are needed to clarify how rural context relates to symptom recognition, healthcare-seeking, referral, and RA diagnosis.

## 1. Introduction

Rheumatoid arthritis (RA) is a chronic systemic inflammatory disease in which early recognition and timely initiation of disease-modifying antirheumatic drugs are important for preventing structural joint damage, functional decline, and impaired quality of life [1,2]. The 2010 American College of Rheumatology/European League Against Rheumatism classification criteria incorporate joint involvement, serological findings, acute-phase reactants, and symptom duration [3]. However, recognizing RA in older adults can be particularly challenging. Elderly-onset rheumatoid arthritis (EORA) is clinically heterogeneous and may present not only with typical small-joint involvement but also with large-joint symptoms, proximal pain, constitutional manifestations, or polymyalgia rheumatica-like features [4,5]. Moreover, musculoskeletal symptoms in older adults may be attributed to aging, osteoarthritis, physical work, or coexisting illnesses, potentially complicating both symptom recognition and clinical assessment [6,7].

Generalist and primary care clinicians often play an important role in the initial evaluation of older adults with newly developed musculoskeletal symptoms, particularly in areas where access to rheumatology services is limited. Previous studies of RA in rural populations have primarily focused on healthcare accessibility, service utilization, travel burden, referral to specialist care, and clinical outcomes [8,9]. Geographic barriers, including the distance and burden associated with traveling to primary or specialist care, may influence when and where patients seek medical attention [9]. Among older adults, healthcare-seeking may also be shaped by family support, social relationships, transportation availability, local healthcare resources, and individual perceptions of symptom severity or meaning [10,11]. These factors may be particularly relevant when early musculoskeletal symptoms cause only limited disruption to daily activities and are therefore tolerated, self-managed, or attributed to normal aging.

Rural context may therefore influence RA diagnosis through several interacting pathways. At the patient level, symptom recognition and interpretation may affect the decision to seek care. At the healthcare-system level, geographic accessibility, the availability of primary and specialist services, and referral pathways may influence when inflammatory arthritis is evaluated. At the clinician level, heterogeneous presentations of EORA may affect recognition of possible RA, particularly when typical peripheral joint manifestations are absent. Previous rural RA research has largely examined whether these factors contribute to delayed access, differences in healthcare utilization, or poorer outcomes [8,9,10,11]. In contrast, considerably less is known about whether the broader geographic context in which patients live is associated with the pattern of joint symptoms documented when RA is ultimately recognized and diagnosed.

This question is particularly relevant in Japan, where geographic maldistribution of physicians remains an important healthcare challenge. Although the overall physician workforce has increased, rural communities have historically had fewer physicians relative to population size, and geographic disparities in physician distribution have persisted [12,13,14]. Such disparities may influence not only access to rheumatology care but also the pathway through which inflammatory arthritis is initially recognized, assessed, and referred. In this context, area-level rurality may capture aspects of the healthcare environment that are not adequately represented by individual patient characteristics alone.

In our previous study of older patients with RA living in a rural region of Japan, older age, social isolation, and wrist symptoms were associated with a longer interval from symptom onset to the study-hospital consultation [15]. That study primarily examined individual-level factors associated with healthcare-seeking and consultation delay. However, it did not investigate whether the broader geographic context of rurality was associated with the pattern of musculoskeletal symptoms documented at the time of RA diagnosis. The present study therefore addresses a related but distinct health-services question by treating geographic rurality as an area-level exposure and documented joint symptom presentation at diagnosis as the clinical outcome.

The Rurality Index for Japan (RIJ) was developed as a postal code-based measure of rurality for healthcare research and incorporates contextual characteristics such as population density, distance to the nearest hospital, remote island status, and weather-related access barriers [16,17]. Compared with conventional rural–urban classifications, the RIJ enables rurality to be examined as a continuous and multidimensional contextual characteristic [16,17]. This approach may be particularly useful when investigating diagnostic pathways because patients living within nominally “rural” regions may nevertheless experience substantially different degrees of geographic isolation and healthcare accessibility.

Accordingly, this study examined the association between RIJ-measured area-level rurality and documented hand, wrist, foot, or ankle symptoms at the time of RA diagnosis among adults aged 65 years or older in rural Japan. We also explored differences in the interval from symptom onset to the study-hospital consultation according to documented joint symptom presentation. We hypothesized that greater area-level rurality would be associated with differences in the joint symptom patterns documented at diagnosis. Importantly, we did not interpret such an association as evidence of a distinct biological RA phenotype according to place of residence; rather, we considered it a potential reflection of differences in symptom recognition, healthcare-seeking, referral, clinical assessment, and documentation within rural diagnostic pathways.

## 2. Materials and Methods

### 2.1. Study Design and Setting

We conducted a cross-sectional analysis of a retrospectively assembled single-center cohort at Unnan City Hospital, a community hospital in mountainous Shimane Prefecture, Japan. The local healthcare environment is characterized by an aging population and potential barriers related to distance, transportation, weather, and the distribution of healthcare resources.

This analysis addressed a health-services question distinct from our previous report using the same cohort [15]: the primary exposure was postal code-based area-level rurality, and the primary outcome was the joint symptom pattern documented at the time of RA diagnosis.

### 2.2. Participants

Eligible participants were patients aged 65 years or older who were diagnosed with RA at Unnan City Hospital between 1 April 2016, and 31 March 2021. During this period, 30,040 patients visited the outpatient department of the hospital. Of these, 3760 patients aged younger than 65 years were excluded, leaving 26,280 patients aged 65 years or older. Among these patients, 26,059 were excluded because they had diagnoses other than RA, resulting in an original cohort of 221 patients diagnosed with RA.

RA diagnosis was confirmed using the 2010 American College of Rheumatology/European League Against Rheumatism classification criteria [3] together with specialist clinical assessment. When the classification score alone was insufficient, radiographic bone erosions compatible with RA supported the diagnosis. Alternative conditions that could better explain the inflammatory musculoskeletal presentation—including calcium pyrophosphate deposition disease, polymyalgia rheumatica, remitting seronegative symmetrical synovitis with pitting edema syndrome, and malignancy-associated inflammatory arthritis—were clinically excluded based on the documented history, physical examination, laboratory and imaging findings, and longitudinal clinical assessment.

For the present secondary analysis, residential postal codes were linked to RIJ scores. All 221 patients in the original RA cohort had residential postal-code information that could be linked to an RIJ score and had data available for the primary outcome. Therefore, no additional patients were excluded, and all 221 patients were included in the final analytic sample.

### 2.3. Data Collection

We retrospectively extracted clinical and demographic data from the electronic medical records. One physician investigator (R.O.) performed the medical-record review and data extraction using predefined variables and anatomical categories established before the present analysis. Extracted variables included age, sex, body mass index, educational history, alcohol use, smoking history, social isolation, medication count, comorbidities, Charlson Comorbidity Index (CCI), and laboratory findings [18].

Laboratory variables included C-reactive protein (CRP), erythrocyte sedimentation rate, rheumatoid factor, anti-cyclic citrullinated peptide (anti-CCP) antibody, hemoglobin, albumin, estimated glomerular filtration rate, creatinine, white blood cell count, and lactate dehydrogenase.

We extracted musculoskeletal symptoms documented at the time of RA diagnosis according to predefined anatomical locations: hand, wrist, elbow, shoulder, neck, hip, knee, ankle, and foot. A location was coded as symptomatic when a musculoskeletal symptom at that anatomical site was explicitly documented in the medical record. If no symptom was documented for a particular location, that location was coded as having no documented symptom. Therefore, the retrospective data could not always distinguish a truly absent symptom from one that was not elicited, recognized, or recorded.

Symptom ascertainment was based on routine clinical documentation and did not reflect a standardized tender or swollen joint count, systematic joint examination, or imaging-confirmed synovitis assessment. Because a single investigator conducted the record review, inter-observer reproducibility was not assessed. The reviewer was not formally blinded to all information that could ultimately be linked to the RIJ; however, symptom coding was based on predefined anatomical categories and explicit clinical documentation rather than interpretation of the RIJ value itself. These features of the retrospective assessment are acknowledged as potential sources of classification and documentation bias.

We calculated the interval from symptom onset to the first documented consultation at the study hospital in days. The date of symptom onset was determined retrospectively from physicians’ documentation of the history of the initial musculoskeletal symptoms in the electronic medical record, and the consultation date was defined as the first documented visit to Unnan City Hospital in the clinical course that ultimately led to the RA diagnosis. Information on earlier consultations at other medical institutions was not comprehensively available; therefore, this variable does not necessarily represent the first healthcare contact attributable to the symptoms [15]. No additional medical records from outside institutions were obtained for the present analysis.

### 2.4. Exposure: Rurality Index for Japan

The primary exposure was area-level rurality measured using the RIJ, a postal code-based measure developed for healthcare research in Japan that incorporates population density, distance to the nearest hospital, remote island status, and weather-related access barriers [16,17]. For the present analysis, we used RIJ2024, the updated version of the RIJ based primarily on nationwide administrative and geographic data corresponding to 2024. Each participant’s RIJ score was assigned using the residential postal code recorded at the time of the relevant hospital visit. Residential postal codes were linked to RIJ scores and were used only for RIJ assignment. We analyzed RIJ as a continuous variable and estimated odds ratios (ORs) per 10-point increase, with higher values indicating greater area-level rurality.

### 2.5. Outcome Measures

The primary outcome was documentation of symptoms involving at least one of the hand, wrist, foot, or ankle locations at RA diagnosis. These locations were combined as a pragmatic measure of peripheral musculoskeletal presentation that could be consistently identified from routine clinical documentation. The composite was not intended to represent a homogeneous anatomical unit, standardized joint count, objective synovitis, or a distinct biological RA phenotype. In particular, the hand and foot comprise multiple joints and anatomically heterogeneous structures; therefore, the composite should be interpreted as a documentation-based measure of clinical presentation rather than evidence of anatomically uniform joint involvement.

We considered a location symptomatic when a musculoskeletal symptom at that anatomical site was explicitly documented in the medical record. The outcome was based on routine clinical documentation and did not require standardized tender or swollen joint counts, systematic joint examination, or imaging-confirmed synovitis. Accordingly, throughout the manuscript we use the term “documented symptoms” rather than “joint involvement” when referring to this outcome.

To examine whether a particular anatomical location drove the composite association, prespecified secondary analyses evaluated the hand, wrist, foot, and ankle separately, as well as other recorded anatomical locations. We also examined documented hand, wrist, or foot symptoms and the total number of documented symptomatic locations. These secondary analyses were considered exploratory.

The interval from symptom onset to the first documented consultation at the study hospital was additionally evaluated as a contextual measure of the pre-diagnostic care pathway. Because earlier consultations at other medical institutions could not be comprehensively ascertained and because consultation timing could lie on the pathway between rural context and presentation at diagnosis, models including this variable were considered exploratory rather than evidence of an independent biological effect of rurality.

### 2.6. Statistical Analysis

Continuous variables were summarized as means with standard deviations or medians with interquartile ranges, according to their distributions, and categorical variables as frequencies and percentages. Characteristics were compared according to the primary outcome using parametric or nonparametric tests for continuous variables and the chi-square or Fisher’s exact test for categorical variables, as appropriate. RIJ scores were compared between groups using the Mann–Whitney U test.

Logistic regression was used to examine the association between RIJ and the primary outcome, with odds ratios (ORs) and 95% confidence intervals (CIs) estimated per 10-point increase in RIJ. The regression models were specified sequentially according to the conceptual roles of the covariates. Model 1 included RIJ alone. Model 2 was considered the primary confounder-adjusted model and included RIJ, age, sex, and Charlson Comorbidity Index (CCI). Age, sex, and comorbidity burden were selected a priori because they may plausibly be associated with both residential context and the recognition or documentation of musculoskeletal symptoms among older adults.

Models 3 and 4 were considered exploratory sequential models rather than progressively more definitive estimates of an independent effect of rurality. Model 3 also included the interval from symptom onset to the study-hospital consultation. Because consultation timing may lie on the pathway between rural context and the clinical presentation documented at diagnosis, it was considered a potential intermediate factor rather than a conventional confounder. Model 3 therefore examined whether the RIJ estimate changed substantially after accounting for consultation timing and was not interpreted as estimating a direct causal effect of rurality.

Model 4 additionally included CRP and anti-CCP antibody levels as disease-related clinical characteristics. We included these variables to explore whether differences in inflammatory activity and serological characteristics materially altered the RIJ estimate; we did not consider them established confounders of the relationship between area-level rurality and documented symptom presentation. Consequently, Models 3 and 4 were interpreted as exploratory analyses.

Because this study was a secondary analysis of an existing cohort, we did not perform an a priori sample size calculation, and we included all eligible participants. The primary outcome occurred in 106 of 221 participants. All variables required for Models 1–4 were available for all 221 participants. The primary adjusted model contained four regression parameters, corresponding to approximately 26.5 outcome events per parameter, whereas the most extensively adjusted model contained seven regression parameters, corresponding to approximately 15.1 outcome events per parameter. These event counts were considered adequate for the planned models, while recognizing the modest overall sample size. A retrospective post hoc power calculation was not performed because effect estimates and their 95% CIs were considered more informative measures of statistical precision.

RIJ was initially modeled as a continuous variable, and effect estimates were reported per 10-point increase to facilitate interpretation. RIJ scores ranged from 24 to 88 in the study population. The linearity assumption for RIJ was assessed in the primary confounder-adjusted model by comparing a model containing RIJ as a linear term with a model in which RIJ was modeled using a natural cubic spline with 3 degrees of freedom, while retaining adjustment for age, sex, and CCI. Model fit was compared using a likelihood-ratio test. This analysis suggested some departure from strict linearity; therefore, the OR per 10-point increase was interpreted as a summary measure of the average association across the observed RIJ range rather than as assuming a constant effect throughout the full range. We also examined the distribution of the primary outcome across RIJ quartiles descriptively as a sensitivity analysis.

Because RIJ was assigned according to residential postal code, potential within-area correlation was considered. Participants represented 76 distinct residential postal-code areas, with a median of 2 participants per area (interquartile range, 1–3; maximum, 14). As a sensitivity analysis, the primary adjusted logistic regression was repeated using standard errors clustered by residential postal-code area.

We examined secondary binary outcomes, including documented hand, wrist, or foot symptoms and individual anatomical symptom locations, using logistic regression. We analyzed the number of documented symptomatic anatomical locations using Poisson regression. We assessed overdispersion using the Pearson dispersion statistic; because we found no evidence of overdispersion, negative binomial regression was not required. All secondary analyses were considered exploratory, and no adjustment for multiple comparisons was performed.

The study-hospital consultation interval, CRP, and anti-CCP antibody levels were entered into the regression models as continuous variables on their original measurement scales without transformation. Anti-CCP values recorded as 0 U/mL were retained as observed laboratory values and were not treated as missing. No imputation was performed because all variables required for Models 1–4 were complete for all 221 participants.

Tests were two-sided, with *p* < 0.05 considered statistically significant. Statistical analyses were performed using EZR (version 1.70) (Saitama Medical Center, Jichi Medical University, Saitama, Japan), a graphical user interface for R [19]. Additional sensitivity analyses evaluating the functional form of RIJ and geographic clustering were performed using R 4.6.1.

### 2.7. Reporting and Ethical Considerations

The study was conducted in accordance with the Declaration of Helsinki and the Ethical Guidelines for Medical and Biological Research Involving Human Subjects in Japan. The study was reported in accordance with the Strengthening the Reporting of Observational Studies in Epidemiology (STROBE) Statement [20]. The Ethics Committee of Unnan City Hospital approved the study protocol (approval number: 20260012). The requirement for written informed consent was waived because of the retrospective observational design, and an opt-out procedure was used. Patients who declined the use of their data were excluded. Direct identifiers were removed before analysis, and residential postal codes were not retained after assignment of the Rurality Index for Japan.

During manuscript preparation, we used generative AI for language editing, manuscript organization, and exploratory cross-checking of tabulated results. However, the authors independently verified all final statistical analyses and numerical results reported in the Abstract, Section 3, Tables, and Supplementary Tables using EZR/R against the original analytic dataset.

## 3. Results

### 3.1. Participant Selection

**Figure 1:**
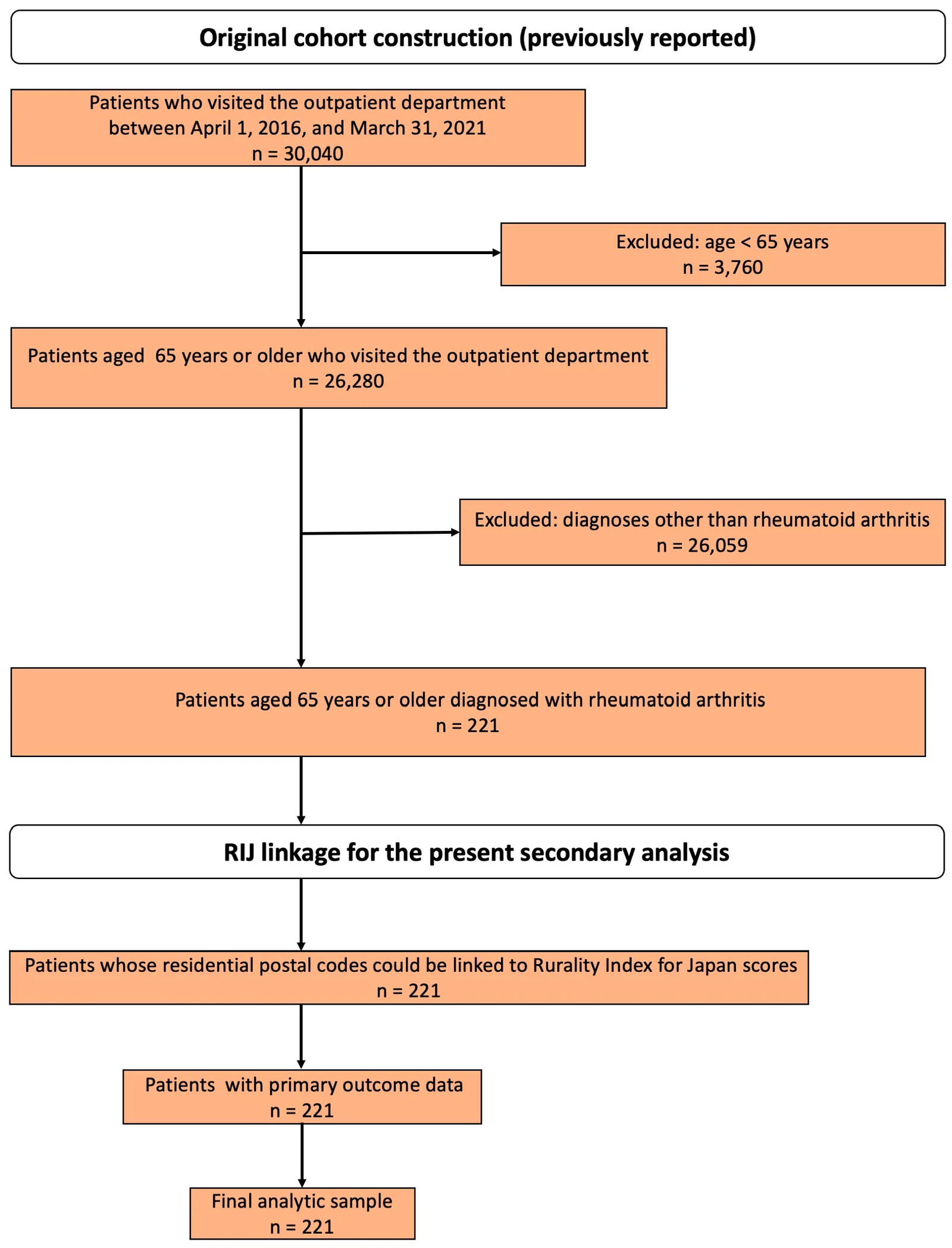
Participant selection flow diagram.

The participant selection process is shown in Figure 1. During the study period, 30,040 patients visited the outpatient department of Unnan City Hospital. After excluding 3760 patients aged younger than 65 years, 26,280 patients aged 65 years or older remained. Among them, 26,059 patients with diagnoses other than RA were excluded, resulting in an original cohort of 221 patients diagnosed with RA. All 221 patients had residential postal codes that could be linked to RIJ scores and had data available for the primary outcome. Therefore, no additional patients were excluded from the present secondary analysis, and all 221 patients were included in the final analytic sample (Figure 1).

Figure legend: During the study period, 30,040 patients visited the outpatient department of Unnan City Hospital. After excluding 3760 patients younger than 65 years and 26,059 patients aged 65 years or older with diagnoses other than rheumatoid arthritis, the original cohort comprised 221 patients aged 65 years or older with rheumatoid arthritis. All 221 patients had residential postal codes that could be linked to Rurality Index for Japan scores and had data available for the primary outcome; therefore, all 221 patients were included in the final analytic sample for the present secondary analysis. RIJ, Rurality Index for Japan.

### 3.2. Patient Characteristics

The mean age was 80.8 years, and 159 patients (71.9%) were female. Documented hand, wrist, foot, or ankle symptoms at diagnosis were present in 106 patients (48.0%); 115 patients (52.0%) had no symptoms documented at these locations. Patients with documented hand, wrist, foot, or ankle symptoms had higher RIJ scores than those without these symptoms (median 75.5 [IQR 58.0–83.0] vs. 66.0 [55.0–76.0]; *p* = 0.004). The interval from symptom onset to the first documented consultation at the study hospital was longer among patients with documented hand, wrist, foot, or ankle symptoms (median 48.5 days [IQR 29.0–82.2] vs. 28.0 days [15.0–56.5]; *p* < 0.001). Patients with these documented symptoms had lower CRP levels (median 1.5 vs. 4.8 mg/dL; *p* < 0.001) and erythrocyte sedimentation rates (52.0 vs. 68.0 mm/h; *p* < 0.001). Rheumatoid factor and anti-CCP antibody levels were higher among patients with documented hand, wrist, foot, or ankle symptoms. The median anti-CCP antibody level in the overall cohort was 0.0 U/mL. Patients without documented symptoms at these locations had a higher CCI and a higher proportion with a history of cancer. Baseline characteristics are shown in Table 1.

### 3.3. Association Between RIJ Score and Documented Hand, Wrist, Foot, or Ankle Symptoms

All 221 participants had complete data for the variables included in Models 1–4; therefore, each regression model included 221 participants, including 106 participants with the primary outcome. RIJ scores ranged from 24 to 88, with a mean of 67.3 ± 14.8, a median of 68, and an interquartile range of 55–79. In the unadjusted logistic regression model, each 10-point increase in RIJ was associated with higher odds of documented hand, wrist, foot, or ankle symptoms at diagnosis (OR 1.28, 95% CI 1.06–1.54; *p* = 0.009). The estimate was similar in the primary confounder-adjusted model including age, sex, and CCI (OR 1.30, 95% CI 1.07–1.57; *p* = 0.008).

In the exploratory sequential models, the RIJ estimate remained similar after additionally incorporating the study-hospital consultation interval (OR 1.29, 95% CI 1.06–1.56; *p* = 0.010) and after further inclusion of CRP and anti-CCP antibody levels (OR 1.29, 95% CI 1.06–1.57; *p* = 0.012) (Table 2). These latter models were interpreted as exploratory because consultation timing may represent an intermediate factor and CRP and anti-CCP levels were included as disease-related clinical characteristics rather than established confounders.

Assessment of the functional form of RIJ suggested some departure from strict linearity (likelihood-ratio *p* = 0.021). In the primary confounder-adjusted model, we assessed this by comparing a model with RIJ as a linear term with a model in which RIJ was modeled using a natural cubic spline with 3 degrees of freedom, while retaining adjustment for age, sex, and CCI. The fitted spline-based association with 95% confidence intervals is shown in Supplementary Figure S1. Accordingly, the OR per 10-point increase summarizes the average association across the observed RIJ range. Descriptively, the proportion of participants with the primary outcome increased across RIJ quartiles: 34.5% (20/58) in the lowest quartile, 44.4% (24/54) in the second quartile, 50.0% (28/56) in the third quartile, and 64.2% (34/53) in the highest quartile.

The 221 participants resided in 76 distinct postal-code areas. The median number of participants per postal-code area was 2 (interquartile range, 1–3), with a maximum of 14. In a sensitivity analysis using standard errors clustered by residential postal-code area, the association in the primary adjusted model remained similar in magnitude (OR per 10-point increase in RIJ, 1.30; 95% CI 1.04–1.61; *p* = 0.020).

### 3.4. Secondary Analyses

Documented hand, wrist, or foot symptoms were present in 105 of 221 participants (47.5%). Each 10-point increase in RIJ was associated with higher odds of documented hand, wrist, or foot symptoms in the unadjusted model (OR 1.26, 95% CI 1.05–1.51; *p* = 0.015) and in the primary adjusted model including age, sex, and CCI (OR 1.27, 95% CI 1.05–1.54; *p* = 0.014). Similar estimates were observed in exploratory models additionally incorporating consultation interval (OR 1.26, 95% CI 1.04–1.53; *p* = 0.018) and CRP and anti-CCP antibody levels (OR 1.26, 95% CI 1.03–1.53; *p* = 0.021) (Table 3).

We additionally examined individual anatomical symptom locations. Documented symptoms were present in the hand in 61 participants, wrist in 56, foot in 23, ankle in 11, elbow in 22, shoulder in 69, neck in 29, hip in 41, and knee in 33. In unadjusted analyses, the estimated ORs per 10-point increase in RIJ ranged from 0.84 for neck symptoms to 1.23 for ankle and foot symptoms. After adjustment for age, sex, and CCI, hand symptoms showed a modest association with RIJ (OR 1.25, 95% CI 1.00–1.56; *p* = 0.049), whereas associations for the other individual anatomical locations were not statistically significant. This finding should be interpreted cautiously because these analyses were exploratory and involved multiple anatomical outcomes (Supplementary Table S1).

The number of documented symptomatic anatomical locations ranged from 0 to 7, with a median of 1 [interquartile range, 1–2]. The distribution showed no evidence of overdispersion; therefore, Poisson regression was used. RIJ was not associated with the number of documented symptomatic locations in the unadjusted model (IRR 1.03 per 10-point increase, 95% CI 0.95–1.10; *p* = 0.494) or the primary adjusted model (IRR 1.02, 95% CI 0.95–1.09; *p* = 0.648). Exploratory sequential adjustment for consultation interval and subsequently CRP and anti-CCP antibody levels produced similar estimates (Supplementary Table S2).

## 4. Discussion

### 4.1. Principal Findings

In this cross-sectional analysis, greater area-level rurality was associated with documentation of hand, wrist, foot, or ankle symptoms at RA diagnosis among older adults treated in a rural healthcare system. The association was modest and remained similar in the primary confounder-adjusted model and in exploratory sequential models incorporating consultation timing, inflammatory markers, and anti-CCP antibody levels. Patients with these documented symptoms also had a longer interval from symptom onset to the study-hospital consultation. Secondary analyses did not identify a single anatomical symptom location that consistently explained the composite association, and RIJ was not associated with the total number of documented symptomatic locations. In addition, accounting for clustering within residential postal-code areas did not materially change the magnitude of the primary association. These findings should be interpreted as characteristics of the documented diagnostic presentation and healthcare pathway, rather than as objective measures of synovitis or evidence that rurality produces a distinct biological RA phenotype.

This analysis extends our previous report from the same cohort in a specific way. The previous study examined individual-level factors associated with consultation delay [15], whereas the present study evaluates an area-level geographic exposure and the symptom pattern documented at diagnosis. The findings therefore address how rural context may be related to the pathway through which RA becomes clinically visible within a local healthcare system. Importantly, the present analysis does not establish that geographic rurality alters RA pathophysiology; rather, it identifies an association that may arise through interacting processes of symptom recognition, healthcare-seeking, referral, clinical assessment, and documentation.

### 4.2. Comparison with Previous Studies

Elderly-onset RA is clinically heterogeneous, with variable joint distributions, polymyalgia rheumatica-like presentations, and frequent seronegative disease [21,22,23]. Such heterogeneity complicates recognition in community practice, particularly among older adults in whom musculoskeletal symptoms may coexist with osteoarthritis, physical strain, multimorbidity, or other age-related conditions. In this cohort, diagnoses were confirmed using classification criteria and specialist assessment, with radiographic erosions used when needed and major alternative diagnoses clinically excluded. Nevertheless, the observed differences in documented symptom patterns should not be interpreted as evidence that rurality is associated with seronegative RA or another biological subtype.

Previous studies show that symptom recognition, perceived severity, and interpretation influence healthcare-seeking in early RA [24,25,26,27], while patients may self-manage symptoms, consult others, or delay care while judging symptom significance [24,28]. These processes are particularly relevant to the present findings because the primary outcome was based on symptoms documented when RA was diagnosed rather than on prospectively standardized joint examination. Symptoms involving the hands, wrists, feet, or ankles may become sufficiently noticeable to prompt consultation or may be more readily interpreted by patients and clinicians as musculoskeletal problems requiring evaluation. However, this explanation remains hypothetical because symptom appraisal itself was not directly measured in the present study.

Research on rurality and RA has largely focused on travel burden, healthcare utilization, diagnostic delay, and outcomes [8,9,29,30,31]. Our findings add a complementary observation: among patients who ultimately received an RA diagnosis at one rural center, area-level rurality was associated with the pattern of symptoms documented at that point in the diagnostic pathway. This does not imply that rurality determines which joints are biologically affected. Rather, geographic context may influence which manifestations are noticed, tolerated, reported, clinically recognized, referred, or documented before a definitive diagnosis is reached.

The secondary analyses provide additional context for interpreting the primary association. Although hand symptoms showed a modest association with RIJ in the primary adjusted analysis, the corresponding associations for other individual anatomical locations were not consistently statistically significant, and RIJ was not associated with the total number of documented symptomatic locations. Because these analyses were exploratory and involved multiple anatomical comparisons, the hand-specific finding should be interpreted cautiously. Collectively, these results do not suggest that the main finding is explained by one specific anatomical manifestation or by a generally greater burden of symptomatic sites in more rural areas. Instead, they reinforce the interpretation of the primary composite outcome as a feature of the documented clinical presentation.

This perspective is relevant in Japan, where definitions of rurality have varied across studies [17,32] and physician maldistribution remains a healthcare challenge [12,13,14,33]. The RIJ provides a standardized, multidimensional measure of area-level rural context [16,17], enabling geographic healthcare research beyond a simple rural–urban dichotomy. In the present cohort, the positive gradient in the proportion of participants with the primary outcome across RIJ quartiles supported the overall direction of the association, although the relationship showed some departure from strict linearity. Accordingly, the OR per 10-point increase in RIJ should be interpreted as a summary of the association across the observed RIJ range rather than as evidence of a constant linear effect at every level of rurality.

### 4.3. Interpretation in the Healthcare Context

Several non-biological mechanisms could generate the observed association. Older adults in more rural areas may seek care when hand, wrist, foot, or ankle symptoms become sufficiently noticeable or interfere with mobility, household work, farming, or self-care. These symptoms may also be more readily recognized as possible inflammatory arthritis by community clinicians, whereas proximal or systemic presentations may create greater diagnostic uncertainty [9,34,35]. However, the present study did not directly measure the effects of mobility limitation, household activities, farming, self-care, symptom appraisal, or clinician recognition. These mechanisms should therefore be regarded as hypotheses rather than explanations demonstrated by our data.

The longer interval from symptom onset to the study-hospital consultation interval among patients with documented hand, wrist, foot, or ankle symptoms also warrants specific consideration. Our previous analysis of the same cohort identified individual-level factors associated with delayed consultation [15]. In the present study, consultation timing was evaluated in relation to documented symptom presentation and was not treated as a conventional confounder because it may lie on the pathway between rural context and the clinical presentation observed at diagnosis. The interval may reflect several overlapping processes, including patients’ interpretation and tolerance of symptoms, decisions to seek medical attention, availability of transportation and local healthcare resources, and the clinical evolution of symptoms before consultation.

Moreover, the interval from symptom onset to the study-hospital consultation should not be equated with the entire diagnostic interval. We documented clinical characteristics at the time of RA diagnosis, whereas the consultation interval ended at the study-hospital consultation recorded in the available data. Therefore, subsequent referral processes, diagnostic assessment, and the time between initial consultation and definitive RA diagnosis may also have influenced the presentation ultimately documented at diagnosis. This distinction matters because rural diagnostic pathways may involve interactions among patient delay, primary care assessment, referral patterns, specialist availability, and local healthcare organization.

We also observed several clinical and laboratory differences between patients with and without documented hand, wrist, foot, or ankle symptoms. Patients with these symptoms had lower CRP and erythrocyte sedimentation rates but higher rheumatoid factor and anti-CCP antibody levels, whereas those without these documented symptoms had a greater comorbidity burden and more frequent cancer history. These differences should be interpreted cautiously because they were not the study’s primary focus and may reflect heterogeneous clinical manifestations of elderly-onset RA, differences in referral or diagnostic pathways, or competing comorbid conditions. The exploratory inclusion of CRP and anti-CCP antibody levels in the sequential regression model was therefore intended to assess whether these disease-related characteristics materially altered the RIJ estimate rather than to establish a biological mechanism. The similarity of the RIJ estimate across these models supports the robustness of the observed association but does not establish causality. These proposed pathways were not directly measured in the present study and should therefore be considered hypotheses for future research rather than established explanations for the observed association.

### 4.4. Implications for Healthcare Delivery

For healthcare delivery, the findings support systematic assessment of peripheral musculoskeletal symptoms in older adults with suspected inflammatory disease while emphasizing that absence of hand, wrist, foot, or ankle symptoms does not exclude RA. This is particularly relevant for seronegative or atypical elderly-onset presentations. In rural settings, where generalist and primary care clinicians may provide the initial assessment before rheumatology consultation, attention to both typical peripheral symptoms and atypical proximal, large-joint, or systemic manifestations may reduce the risk of overlooking inflammatory arthritis.

The findings are consistent with the possibility that RA diagnosis may be shaped by multiple stages within a diagnostic pathway, including symptom recognition, healthcare-seeking, referral, and clinical assessment; however, the present study did not directly measure these processes. Geographic barriers, patient interpretation of symptoms, accessibility of local services, referral processes, and clinician recognition may interact before a diagnosis is established. Although this study did not directly measure these processes, the observed association between RIJ and documented symptom presentation provides a rationale for studying them prospectively.

At a systems level, area-based measures such as the RIJ may help characterize communities where access, diagnostic support, referral pathways, or tele-rheumatology resources warrant further study. The present findings do not establish that RIJ can be used for individual clinical prediction, nor do they identify a threshold at which rurality becomes clinically important. Rather, they support investigating rural context as one component of diagnostic pathway research and suggest that future studies should combine area-level rurality with direct individual-level measures of healthcare accessibility, transportation, social support, health literacy, and referral experiences.

### 4.5. Strengths

Strengths include use of a validated area-level rurality measure, complete linkage of the primary exposure and outcome, and detailed clinical data from a real-world community hospital cohort. Sequential models allowed examination of whether the association was materially altered after accounting for demographic characteristics and comorbidity, while consultation timing, inflammatory markers, and anti-CCP antibody levels were evaluated in exploratory models according to their conceptual roles.

Additional sensitivity and secondary analyses strengthened interpretation of the main finding. We examined the functional form of RIJ, evaluated potential clustering among participants living within the same postal-code area, assessed individual anatomical symptom locations, and analyzed the number of documented symptomatic locations. The similarity of the primary effect estimate after accounting for postal-code clustering and the absence of a consistent single anatomical driver provide additional context for interpreting the observed association.

### 4.6. Limitations

This study has several limitations. First, inclusion was restricted to patients who were ultimately diagnosed with RA at a single rural community hospital. Referral patterns, healthcare-seeking behavior, geographic accessibility, and local diagnostic practice may therefore have influenced both cohort entry and symptom documentation. Importantly, both area-level rurality and symptom presentation could potentially affect whether and when patients sought care, were referred, reached the study hospital, and ultimately received an RA diagnosis. Selection into the analytic cohort may therefore have been related to both the exposure and the outcome, potentially influencing the observed association. The findings cannot be generalized to all older adults with inflammatory musculoskeletal symptoms, patients with undiagnosed RA, urban populations, or other healthcare systems, and they do not estimate an association between rurality and the occurrence or biological phenotype of RA.

Second, the sample size was modest and limited the complexity of multivariable modeling and the precision of analyses involving less common anatomical symptoms. Although the number of primary outcome events was sufficient for the prespecified regression models, the individual-location analyses were exploratory and should be interpreted cautiously.

Third, the primary outcome was retrospectively extracted from routine medical records. It did not require standardized tender or swollen joint counts, systematic palpation, or imaging confirmation of active synovitis; documentation and recall bias are therefore possible. In particular, absence of a documented symptom could not always be distinguished from a symptom that was not specifically elicited, recognized, or recorded. Thus, the observed association may reflect differences not only in actual symptom presentation but also in patient reporting, clinician recognition, and documentation practices. If these processes differed according to referral pathway or healthcare context associated with rurality, differential misclassification of the outcome could have influenced the magnitude or direction of the observed association.

In addition, because the cohort consisted of older adults, osteoarthritis and other musculoskeletal disorders may have contributed to symptoms documented at individual anatomical locations. Therefore, documented pain, swelling, stiffness, or tenderness cannot necessarily be attributed exclusively to inflammatory RA activity. These considerations reinforce that the primary outcome should be interpreted as documented clinical presentation rather than objective synovitis or an anatomical RA phenotype.

Fourth, RIJ is an area-level measure and does not directly capture individual transportation, income, health literacy, family support, occupation, physical function, travel time, or access to rheumatologists. Although the primary effect estimate was similar after accounting for clustering within residential postal-code areas, residual contextual confounding remains possible. The association between RIJ and the primary outcome also showed some departure from strict linearity; therefore, the OR per 10-point increase should be interpreted as an average association across the observed RIJ range rather than as a constant effect at all levels of rurality.

Fifth, residual confounding is possible, including effects of local referral practices and clinician experience. Consultation timing may also be an intermediate factor, so models that adjust for it should not be interpreted as isolating a direct or biological effect of rurality. Similarly, CRP and anti-CCP antibody levels were included as disease-related clinical characteristics in exploratory analyses rather than as established confounders.

Sixth, the cross-sectional design precludes temporal or causal inference. The association may reflect selection, healthcare-seeking, referral, symptom recognition, clinical assessment, or documentation processes rather than any effect of rurality on RA manifestations. The present study also could not fully distinguish patient delay, referral delay, diagnostic delay, and symptom progression across the pathway from symptom onset to definitive diagnosis.

Finally, elderly-onset RA remains clinically heterogeneous despite careful diagnostic confirmation and exclusion of major mimicking conditions. Multicenter studies with prospectively standardized joint assessments, inclusion of patients across different stages of the diagnostic pathway, and direct individual-level measures of healthcare accessibility are needed to determine whether the present association is reproducible and to clarify the relative contributions of symptom recognition, healthcare-seeking, referral, and documentation processes.

The consultation interval was based on the first documented consultation at the study hospital, and earlier contacts with other healthcare institutions could not be comprehensively identified. The measure therefore cannot distinguish patient delay from prior healthcare use, referral processes, or access-related delay. Furthermore, because clinical presentation was assessed at RA diagnosis, the unmeasured interval between the first study-hospital consultation and definitive diagnosis may have allowed further symptom evolution or diagnostic processes to influence the documented presentation.

## 5. Conclusions

Among older adults diagnosed with RA at a rural Japanese community hospital, greater area-level rurality was modestly associated with documentation of hand, wrist, foot, or ankle symptoms at diagnosis. This association should be interpreted as a feature of the diagnostic pathway and documented clinical presentation rather than as evidence that rurality produces a distinct biological RA phenotype.

These findings suggest that rural context may be relevant to how inflammatory arthritis becomes clinically recognized and documented, potentially through differences in symptom recognition, healthcare-seeking, referral processes, access to care, and clinical assessment. For clinicians, particularly those practicing in rural settings, systematic assessment of musculoskeletal symptoms remains important, while the absence of hand, wrist, foot, or ankle symptoms should not exclude RA in older adults with other features suggestive of inflammatory disease. Attention to atypical, proximal, large-joint, or systemic presentations may be especially important in elderly-onset RA.

At the healthcare-system level, area-based measures such as the RIJ may be useful for identifying communities in which diagnostic support, referral pathways, rheumatology access, or tele-rheumatology services warrant further evaluation. However, RIJ should not be interpreted as an individual-level clinical prediction tool on the basis of the present findings.

Future research should include prospective multicenter studies with standardized tender and swollen joint assessments and, where appropriate, imaging confirmation of synovitis. Studies should also incorporate direct individual-level measures of healthcare accessibility, including transportation availability, travel time, family and social support, health literacy, symptom appraisal, and access to rheumatology services. More detailed evaluation of the pathway from symptom onset through initial consultation, referral, and definitive diagnosis will be important for clarifying how rural context influences symptom recognition, healthcare-seeking, and RA diagnosis.

## Figures and Tables

**Table 1:** Characteristics of older adults with rheumatoid arthritis according to documented hand, wrist, foot, or ankle symptoms at diagnosis.

Characteristic	Total (n = 221)	Without Documented Hand/Wrist/Foot/Ankle Symptoms (n = 115)	With Documented Hand/Wrist/Foot/Ankle Symptoms (n = 106)	*p* Value
Age, years	80.8 ± 11.3	80.5 ± 12.2	81.2 ± 10.3	0.855
Female sex, n (%)	159 (71.9)	78 (67.8)	81 (76.4)	0.156
BMI, kg/m^2^	21.1 ± 4.0	20.7 ± 3.8	21.6 ± 4.2	0.082
Interval from symptom onset to first documented consultation at the study hospital, days	36.0 [19.0–74.0]	28.0 [15.0–56.5]	48.5 [29.0–82.2]	<0.001
Rurality Index for Japan score	68.0 [55.0–79.0]	66.0 [55.0–76.0]	75.5 [58.0–83.0]	0.004
Charlson Comorbidity Index	4.0 [3.0–4.0]	4.0 [4.0–5.0]	4.0 [3.0–4.0]	0.009
Diabetes mellitus, n (%)	25 (11.3)	14 (12.2)	11 (10.4)	0.674
Chronic kidney disease, n (%)	5 (2.3)	3 (2.6)	2 (1.9)	1.000
Dementia, n (%)	11 (5.0)	9 (7.8)	2 (1.9)	0.061
Cancer history, n (%)	19 (8.6)	17 (14.8)	2 (1.9)	<0.001
CRP, mg/dL	2.7 [0.8–6.6]	4.8 [1.9–8.2]	1.5 [0.3–3.5]	<0.001
ESR, mm/h	57.0 [40.0–85.0]	68.0 [46.5–88.0]	52.0 [30.0–76.0]	<0.001
Rheumatoid factor, IU/mL	12.0 [2.0–73.0]	8.0 [1.0–31.0]	30.0 [3.0–117.2]	0.007
Anti-CCP antibody, U/mL	0.0 [0.0–30.0]	0.0 [0.0–0.0]	1.0 [0.0–113.5]	<0.001

Legend: Values are presented as mean ± standard deviation, median [interquartile range], or n (%), as appropriate. BMI, body mass index; CCI, Charlson Comorbidity Index; CRP, C-reactive protein; ESR, erythrocyte sedimentation rate; RF, rheumatoid factor; anti-CCP, anti-cyclic citrullinated peptide antibody; RIJ, Rurality Index for Japan. kg/m^2^, kilograms per square meter; mg/dL, milligrams per deciliter; mm/h, millimeters per hour; IU/mL, international units per milliliter; U/mL, units per milliliter.

**Table 2:** Logistic regression models for the association between Rurality Index for Japan score and documented hand, wrist, foot, or ankle symptoms at diagnosis.

Model	Adjustments	n	Events	OR per 10-Point Increase in RIJ	95% CI	*p* Value
Model 1	RIJ only	221	106	1.28	1.06–1.54	0.009
Model 2	Age, sex, CCI	221	106	1.30	1.07–1.57	0.008
Model 3	Model 2 + Study-hospital consultation interval	221	106	1.29	1.06–1.56	0.010
Model 4	Model 3 + CRP + anti-CCP antibody	221	106	1.29	1.06–1.57	0.012

Legend: OR, odds ratio; CI, confidence interval; RIJ, Rurality Index for Japan; CCI, Charlson Comorbidity Index; CRP, C-reactive protein; anti-CCP, anti-cyclic citrullinated peptide antibody. ORs for RIJ are expressed per 10-point increase in RIJ score. All models included 221 participants, of whom 106 had the primary outcome. No observations were excluded because of missing covariate data.

**Table 3:** Secondary analysis of the association between Rurality Index for Japan score and documented hand, wrist, or foot symptoms at diagnosis.

Outcome	Model	Adjustments	OR per 10-Point Increase in RIJ	95% CI	*p* Value
Documented hand, wrist, or foot symptoms	Model 1	RIJ only	1.26	1.05–1.51	0.015
Documented hand, wrist, or foot symptoms	Model 2	Age, sex, CCI	1.27	1.05–1.54	0.014
Documented hand, wrist, or foot symptoms	Model 3	Model 2 + duration from symptom onset to the study-hospital consultation interval	1.26	1.04–1.53	0.018
Documented hand, wrist, or foot symptoms	Model 4	Model 3 + CRP + anti-CCP antibody	1.26	1.03–1.53	0.021

Legend: OR, odds ratio; CI, confidence interval; RIJ, Rurality Index for Japan; CCI, Charlson Comorbidity Index; CRP, C-reactive protein; anti-CCP, anti-cyclic citrullinated peptide antibody. ORs for RIJ are expressed per 10-point increase in RIJ score. All models included 221 participants, of whom 105 had the secondary binary outcome. No observations were excluded because of missing covariate data.

## Data Availability

The datasets generated and/or analyzed during the current study are not publicly available because they contain potentially identifiable clinical information from a single rural community. De-identified data may be available from the corresponding author upon reasonable request and with permission from the Ethics Committee of Unnan City Hospital.

## Supplementary Materials

The following supporting information can be downloaded at: https://www.mdpi.com/article/10.3390/healthcare14203453/s1, Table S1. Association between RIJ and documented symptoms at individual anatomical locations; Table S2. Association between RIJ and number of documented symptomatic anatomical locations; Figure S1. Adjusted association between Rurality Index for Japan score and documented hand, wrist, foot, or ankle symptoms at diagnosis using a natural cubic spline.
