# Supplementary material for: Area-Level Rurality and Documented Musculoskeletal Symptom Presentation at Rheumatoid Arthritis Diagnosis Among Older Adults in Rural Japan: A Cross-Sectional Study
Source: Healthcare (Basel). 2026 Oct 15;14(20):3453. doi: 10.3390/healthcare14203453 (PMC13704138; doi:10.3390/healthcare14203453)
Supplement: Supplementary file 1 [file healthcare-14-03453-s001.zip › healthcare-4565736-supplementary.pdf]

Supplementary Table S1. Association between RIJ and documented symptoms at individual anatomical locations

Anatomical location	n with symptom	Model 1 OR (95% CI), p	Model 2 OR (95% CI), p	Model 3 OR (95% CI), p	Model 4 OR (95% CI), p
Hand	61	1.22 (0.99–1.50), 0.065	1.25 (1.00–1.56), 0.049	1.24 (0.99–1.54), 0.062	1.21 (0.97–1.52), 0.095
Wrist	56	1.03 (0.84–1.27), 0.776	1.02 (0.83–1.27), 0.820	1.03 (0.83–1.27), 0.813	1.04 (0.84–1.30), 0.719
Foot	23	1.23 (0.90–1.69), 0.192	1.19 (0.86–1.65), 0.283	1.20 (0.87–1.67), 0.264	1.20 (0.87–1.67), 0.270
Ankle	11	1.23 (0.79–1.91), 0.363	1.20 (0.75–1.90), 0.445	1.17 (0.74–1.86), 0.507	1.15 (0.72–1.85), 0.550
Elbow	22	1.03 (0.77–1.39), 0.834	1.02 (0.75–1.40), 0.884	1.02 (0.75–1.40), 0.880	1.06 (0.78–1.46), 0.700
Shoulder	69	0.88 (0.73–1.06), 0.186	0.87 (0.71–1.06), 0.159	0.87 (0.72–1.06), 0.179	0.88 (0.73–1.08), 0.228
Neck	29	0.84 (0.65–1.08), 0.177	0.84 (0.65–1.08), 0.177	0.84 (0.64–1.08), 0.175	0.84 (0.65–1.10), 0.208
Hip	41	0.99 (0.79–1.25), 0.958	1.01 (0.80–1.27), 0.960	1.03 (0.82–1.31), 0.778	1.07 (0.84–1.36), 0.589
Knee	33	1.13 (0.87–1.46), 0.355	1.11 (0.86–1.44), 0.431	1.07 (0.82–1.39), 0.640	1.04 (0.79–1.36), 0.791

Footnote:

OR, odds ratio; CI, confidence interval; RIJ, Rurality Index for Japan; CCI, Charlson Comorbidity Index. ORs are expressed per 10-point increase in RIJ. Model 1 includes RIJ only. Model 2 adjusts for age, sex, and CCI. Model 3 additionally includes the study-hospital consultation interval. Model 4 additionally includes CRP and anti-CCP antibody levels. All models included 221 participants. Analyses were exploratory, and no adjustment for multiple comparisons was performed.

Supplementary Table S2. Association between RIJ and number of documented symptomatic anatomical locations

Model	Adjustment	IRR per 10-point RIJ	95% CI	p
Model 1	RIJ only	1.03	0.95–1.10	0.494
Model 2	Age, sex, CCI	1.02	0.95–1.09	0.648
Model 3	Model 2 + study-hospital consultation interval	1.01	0.94–1.09	0.696
Model 4	Model 3 + CRP + anti-CCP antibody	1.02	0.95–1.10	0.601

Footnote: IRR, incidence rate ratio; CI, confidence interval; RIJ, Rurality Index for Japan; CCI, Charlson Comorbidity Index; CRP, C-reactive protein; anti-CCP, anti-cyclic citrullinated peptide antibody. IRRs are expressed per 10-point increase in RIJ. Poisson regression was used because no evidence of overdispersion was observed. All models included 221 participants.

Supplementary Figure S1. Adjusted association between Rurality Index for Japan score and documented hand, wrist, foot, or ankle symptoms at diagnosis using a natural cubic spline.

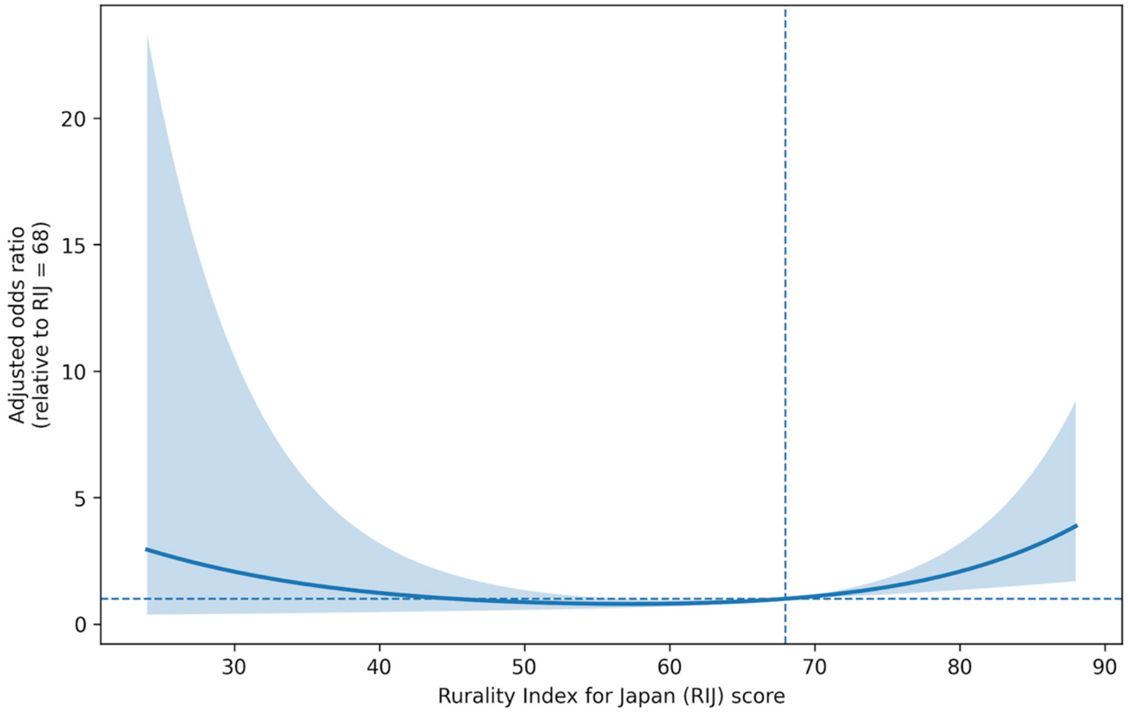

Legend: The solid line represents the fitted odds ratio for documented hand, wrist, foot, or ankle symptoms at diagnosis according to the Rurality Index for Japan (RIJ), estimated from the primary confounder-adjusted logistic regression model in which RIJ was modeled using a natural cubic spline with 3 degrees of freedom. The model was adjusted for age, sex, and Charlson Comorbidity Index (CCI). Shaded bands indicate 95% confidence intervals. Odds ratios are shown relative to the median RIJ score in the cohort (RIJ = 68), and the plot is restricted to the observed RIJ range (24–88). The likelihood-ratio test comparing the spline model with the corresponding linear RIJ model indicated departure from strict linearity ($p = 0.021$).
